# Co-immunomodulatory Activities of Anionic Macromolecules Extracted from *Codium fragile* with Red Ginseng Extract on Peritoneal Macrophage of Immune-Suppressed Mice

**DOI:** 10.4014/jmb.1909.09062

**Published:** 2019-12-30

**Authors:** Ji Eun Kim, Chaiwat Monmai, Weerawan Rod-in, A-yeong Jang, Sang-Guan You, Sang-min Lee, Seok-Kyu Jung, Woo Jung Park

**Affiliations:** 1 Department of Wellness-Bio Industry, Gangneung-Wonju National University, Gangneung 25457, Republic of Korea; 2 Department of Marine Food Science and Technology, Gangneung-Wonju National University, Gangneung 5457, Republic of Korea; 3 Department of Marine Biotechnology, Gangneung-Wonju National University, Gangneung 25457, Republic of Korea; 4 Department of Horticulture, Daegu Catholic University, Gyeongsan 3830, Republic of Korea

**Keywords:** Anionic macromolecule, *Codium fragile*, ginseng, co-immunomodulatory, cyclophosphamide

## Abstract

In this study we investigated the immune effects of oral administration of anionic macromolecules extracted from *Codium fragile* (CFAM) and red ginseng extract mixture on the peritoneal macrophage cells in immune-suppressed mice. Cyclophosphamide (CY) induces the immune-suppressed condition. CY-treated mice were orally fed with different concentrations of CFAM supplemented with red ginseng extract and the peritoneal macrophages collected. CY treatment significantly decreased the immune activities of peritoneal macrophages, compared to the normal mice. The administration of CFAM mixed with red ginseng extract significantly boosted the viability of macrophage cells and nitric oxide production of peritoneal macrophages. Further, the oral administration of CFAM mixed with red ginseng extract up-regulated the expression of *iNOS*, *COX-2*, and *TLR-4* as well as cytokines such as *IL-1*β, *IL*-6, *TNF*-α, and *IFN*-γ more than the red ginseng-treated group. This study showed that CFAM enhanced the immune activity of red ginseng extract in the peritoneal macrophage cells of immune-suppressed mice. Furthermore, CFAM might be used as a co-stimulant of red ginseng extract through the regulation of macrophage cells for the enhancement of human health and immunity.

## Introduction

Innate immunity is the body’s first defense mechanism against pathogens and one of the significant systemic reactions that prevent infection to maintain physiological homeostasis [1, 2]. In this innate immune response, macrophages are important immune cells that perform several roles such as host defense, inflammation control, and remodeling of tissue [3]. Macrophage cells normally can be activated from rest by various stimuli for the purpose of immune response [4]. Importantly, macrophages are some of the first cells to start immune response, which partly depend on their Toll-like and scavenger receptors, to specifically respond against foreign proteins, lipoproteins, polysaccharides, and other molecules, and finally play their role in the elimination of external agents such as microorganisms and viruses that enter the body to cause infections [5].

Most anticancer drugs using chemotherapy strategy have shown negative effects on normal immune cells even though they can remove tumor cells [6]. Cyclophosphamide (CY) is a widely used chemotherapeutic drug. In addition, the antitumor effect of CY such as the death of immunogenic cancer cells is proportional to the dosage of CY, often causing immunosuppression and cytotoxic effects, and high doses of CY can cause lymphopenia and cytokine storms [7, 8]. CY also inhibits humoral immune and cellular immune responses by impeding the differentiation and proliferation of macrophages, as well as T and B mother cells [9,10]. Therefore, it is necessary to find a method for reducing the side effects of CY [11, 12].

Ginseng (*Panax ginseng*) has been one of the most popular and widely consumed traditional medicinal products for over 2,000 years [13] while also being used as a material for health foods in Asian countries [14, 15]. Red ginseng extract has been studied with respect to cancer suppression, blood circulation improvement, and infection-defending action [16]. Various studies have been carried out on the benign reaction of red ginseng on important diseases such as cancer [17], neuronal disease [18], cardiovascular diseases [19], and immune system disorders [20]. Additionally, red ginseng was reported to show antioxidant effects [21] and immune-enhancement effects [22]. *Codium fragile* is an edible green algae widely disseminated in Northern Europe, Oceania, and East Asia [23] and has been studied for its bio-functional activities including carcinogenic, anti-inflammatory and immune-enhancement effects [24, 25]. According to the report by Tabarsa *et al*. [26], anionic macromolecules extracted from *C. fragile* (CFAM) and CFAM provided immune-stimulatory activities in murine macrophage cells. Previous research found that the combination of bioactive materials may give synergetic effects when compared to individual material use [27].

Our recent study showed that CFAM stimulated immune organs such as the spleen and macrophages of CY-induced, immune-suppressed mice [26, 27]. In this study, we investigated the immune effects of oral administration of CFAM and red ginseng extract mixture on the peritoneal macrophage cells in immune-suppressed mice.

## Materials and Methods

### Animals

Inbred male and 6-week-old BALB/c mice weighing between 21 - 23 g were purchased from Central Lab. Animal Inc. (Korea). All the animals were provided with standard laboratory diet and water for one week before starting the experiment. The experiment protocols in this study were approved by the Gangneung-Wonju National University Committee (GWNU-2018-20).

### Materials

Red ginseng extract used is a commercial ginseng syrup extracted from Korean 6-year-old red ginseng purchased from Korea Ginseng Corp. (Korea). CY, levamisole (LVS; an immunostimulant), neutral red solution (0.09% mass fraction of solute), and Griess reagent were obtained from Sigma-Aldrich (USA). LVS induces innate-specific immunity in mammals [28] and is a T cell-independent antigen that modulates natural killer cells and increases both major histocompatibility complex (MHC) receptor expression and cytokine levels [29]. EZ-Cytox Cell Viability Assay Kit was acquired from Daeil Labservice (Korea) and Tri reagent was acquired from Molecular Research Center, Inc. (USA). A high capacity cDNA reverse transcription kit was acquired from Applied Biosystems (USA) and SYBR Premix Ex Taq II were purchased from Takara Bio Inc. (Japan).

### Anionic Macromolecule Extraction

CFAM was extracted as described in our previous research [26]. CFAM was isolated from *C. fragile* by solvents of EtOH and acetone with filtration, centrifugation, evaporation, and deproteinization after milling the samples [30]. The extracted CFAM consisted mainly of carbohydrates (54.6%), proteins (15.7%), sulfates (13.0%) and a small amount of uronic acid (1.4%). Monosaccharide composition analysis revealed that galactose (59.5%) was the major sugar of CFAM.

### Development of Immune-Suppressed Mice and Treatment Protocols

After acclimatization for one week, mice were randomly separated into eight groups (*n* = 5). Two groups were orally administrated with saline (normal group and CY group). The other groups were orally administered with different concentrations of CFAM (0, 25, 50, 75, and 100 mg/kg BW) supplemented with red ginseng extract. LVS was used as a positive control [31] and was orally administered with a concentration of 40 mg/kg BW. All groups were treated one time per day for 10 consecutive days. At day 4-6 after starting administration, all mice, except those in the normal group were given intraperitoneally with CY (80 mg/kg BW), and then sacrificed 24 h after treatment completion.

### Peritoneal Macrophage Preparation

Peritoneal macrophages were prepared using the Ray and Dittel method [32] as previously described [27]. After injection into the peritoneal cavity of each mouse with ice-cold phosphate buffered saline (PBS) supplemented with 3% fetal calf serum (FCS), the injected PBS was recollected and the cell pellet obtained after centrifugation for further experiments.

### Peritoneal Macrophage Viability and Nitric Oxide (NO) Production Assay

Isolated peritoneal macrophages were supplemented with or without 1 μg/ml of lipopolysaccharides (LPS). After 24 h incubation, NO production in the cultured medium was examined using Griess reagent [33]. The treated cells were also used to investigate macrophage viability using the EZ-Cytox Cell Viability Assay Kit [34]. The treated cells were incubated with the dilution of EZ-Cytox Cell Viability solution at 37°C for 1 h, after which the solution was measured using a microplate reader (EL800, BioTek, USA) at the absorbance of 450 nm. Macrophage viability ratio (%) was evaluated using the following equation:

$$\mathrm{Macrophage}\mathrm{viability}\mathrm{ratio}(\%)=\frac{\mathrm{Absorbance}\mathrm{of}\mathrm{test}\mathrm{group}}{\mathrm{Absorbance}\mathrm{of}\mathrm{control}\mathrm{group}}\times 100$$

### Peritoneal Macrophage Phagocytosis Assay

For the evaluation of the phagocytosis of peritoneal macrophages, the neutral red uptake method was tested, as mentioned in previous studies [11, 35]. Phagocytosis of macrophage cells was measured using a microplate reader [absorbance (**A**), 540 nm]. Phagocytosis ratio was assayed using the following equation:

$$\mathrm{Phagocytosis}\mathrm{ratio}(\%)=\frac{\mathrm{Atest}}{\mathrm{Acontrol}}\times 100$$

### RNA Isolation and Reverse Transcription

RNA was gained from the LPS-stimulated peritoneal macrophages by Tri reagent. After confirming the quantity (500 ng) and quality (A_260_/A_280_ ratio = 1.80 – 2.10; The A_260_/A_280_ ratio gives an indication of purity and pure RNA preparation has expected ratios of ≥2.0) of the RNA extracted by a nanophotometer (Implen, Germany), a high capacity cDNA reverse transcription kit was used for the synthesis of cDNA using RNA as a template.

### Immune-Associated Gene Expression Assay

Immune-associated gene expression in peritoneal macrophages was analyzed using SYBR Premix Ex Taq II and a QuantStudio 3 FlexReal-Time PCR System (ThermoFisher Scientific, USA) with 40 cycles of reaction. The specific primer sets for immune-associated genes are presented in Table 1. Relative gene expression was calculated using the 2^-ΔΔCT^ method [36] and β-actin as the reference gene.

### Statistical Analysis

All results were statistically analyzed with Statistix 8.1 Statistics Software (USA) using one-way analysis of variance compared with the control. Thereafter, the Tukey post-hoc test was used to measure the significance at *p* <0.05.

## Results

### Effect of CFAM and Ginseng Mixture on Peritoneal Macrophage Viability and NO Production

**Fig. 1:**
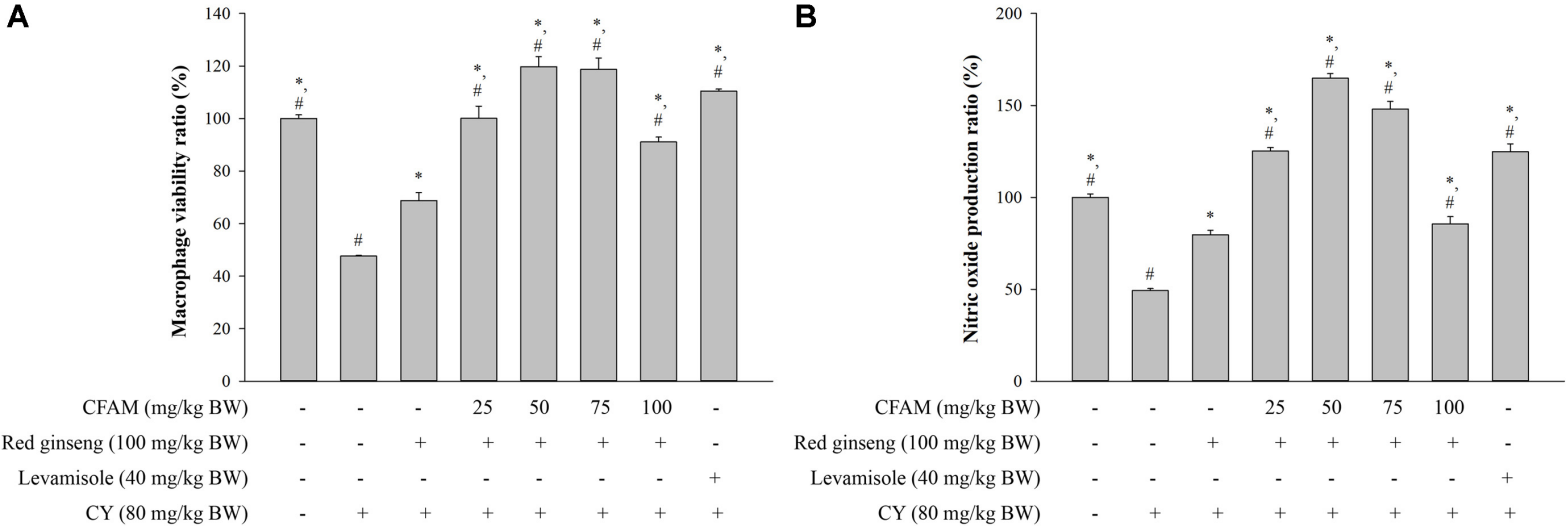
Effects of CFAM and ginseng mixture on peritoneal macrophage. (**A**) Peritoneal macrophage viability, and (**B**) peritoneal macrophage NO production. There was significant difference between CY group (*, *p* < 0.05) and ginseng group (#, *p* < 0.05).

Peritoneal macrophages were collected from each group of mice to examine the effects of CFAM mixed with red ginseng on macrophage viability and NO production in macrophage cells. The treatment of red ginseng extract without CFAM significantly enhanced peritoneal macrophage viability (Fig. 1A) and NO production (Fig. 1B). In addition, red ginseng extract supplemented with CFAM significantly increased macrophage viability and NO production compared to the treatment of red ginseng extract alone. Furthermore, the mixture of CFAM and red ginseng extract restored peritoneal macrophage viability and NO production to the same or higher level than the normal mice in CY-induced immunosuppression condition.

### Effect of CFAM and Ginseng Mixture on Peritoneal Macrophage Phagocytosis

**Fig. 2:**
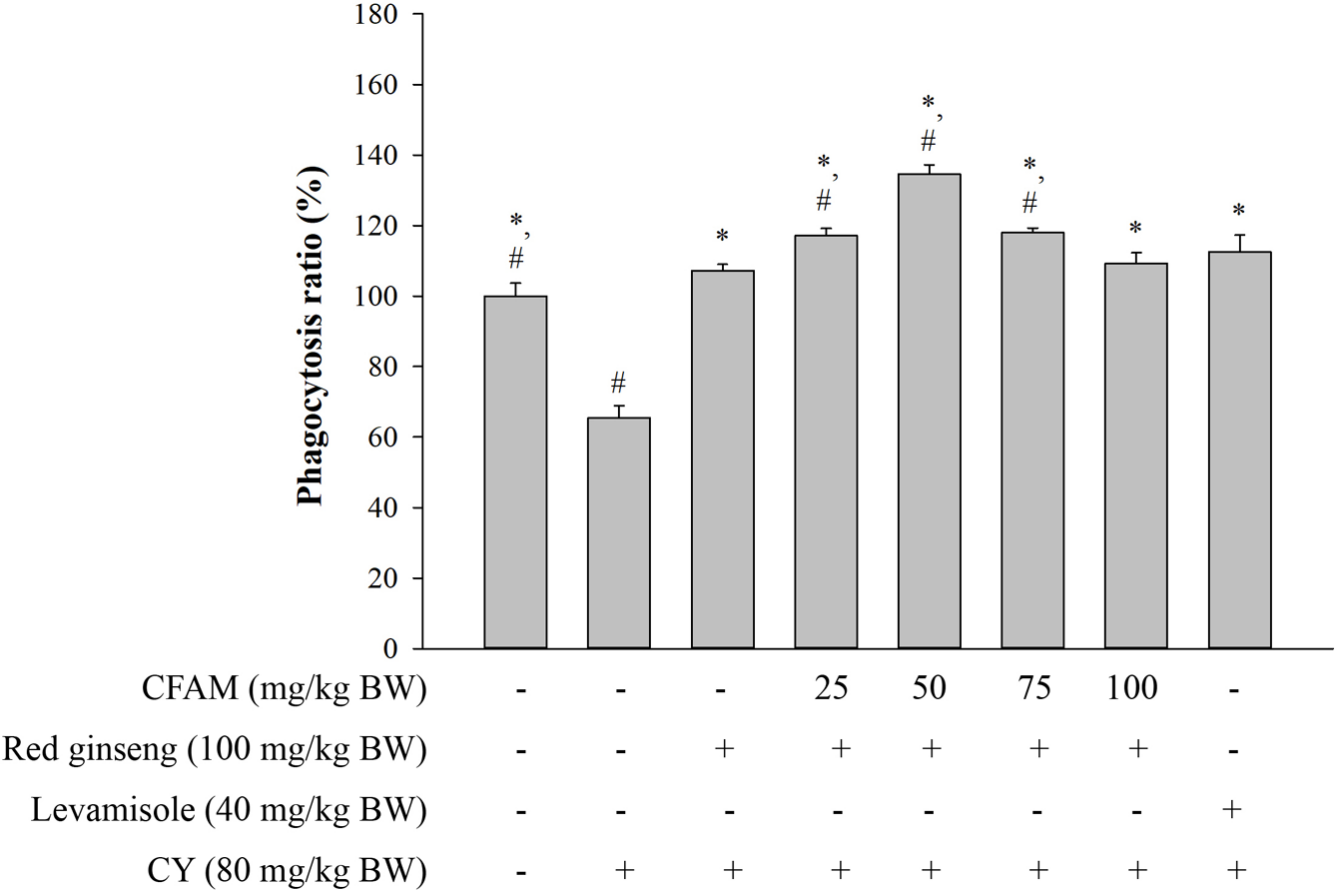
Effects of CFAM and ginseng mixture on peritoneal macrophage phagocytosis ratio. There was significant difference between the CY group (*, *p*<0.05) and ginseng group (#, *p* < 0.05).

As shown in Fig. 2, CY treatment significantly decreased peritoneal macrophage phagocytosis compared to the normal mice. The mixture of CFAM and red ginseng extract significantly increased peritoneal macrophage phagocytosis in the immune-suppressed mice and exhibited recovery of phagocytosis at a ratio similar to or more than normal mice.

### Effect of CFAM and Ginseng Mixture on Peritoneal Macrophage Immune Gene Expression

**Fig. 3:**
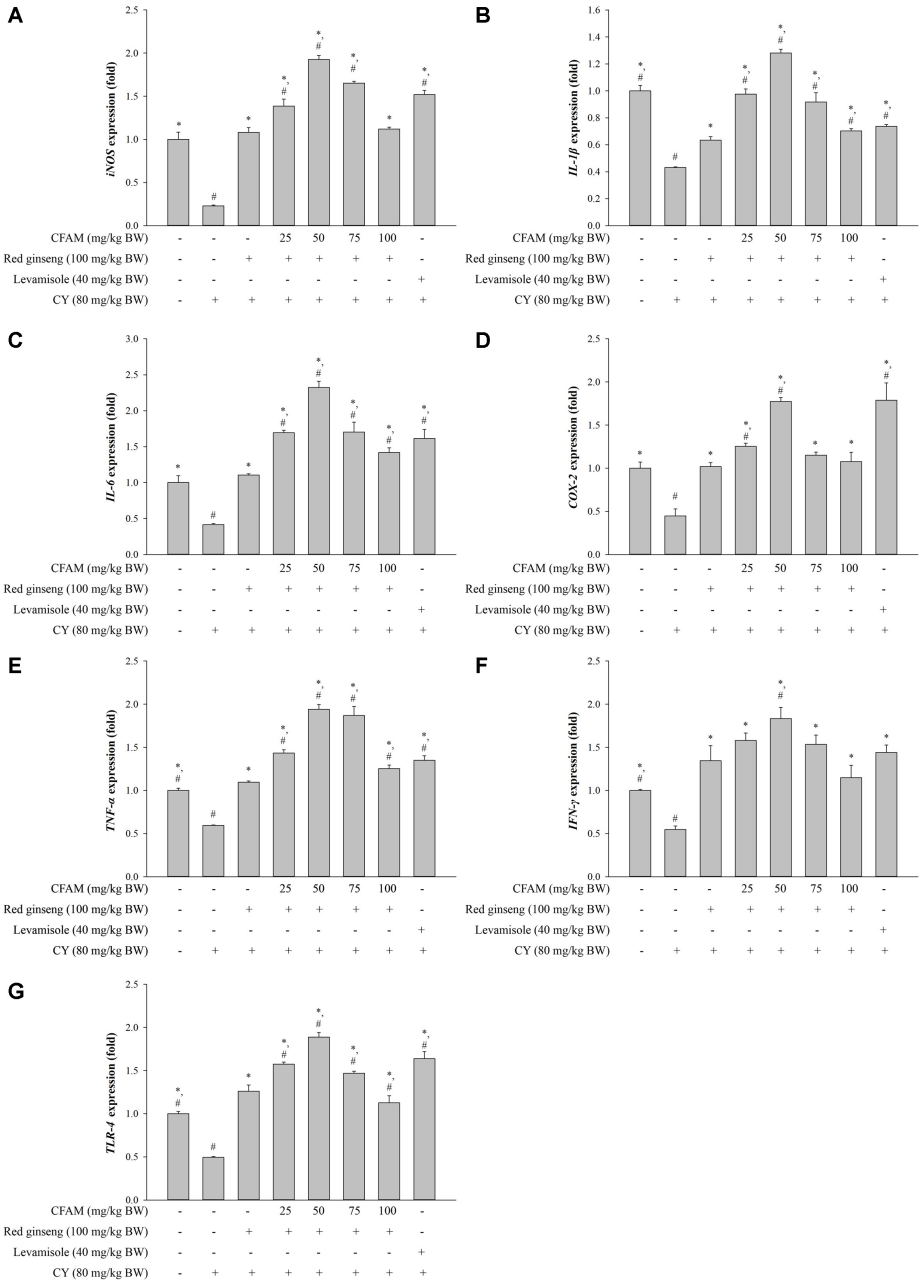
Effects of CFAM and ginseng mixture on peritoneal macrophage immune-associated gene expression. (**A**) *iNOS*, (**B**) *IL-1β*, (**C**) *IL-6*, (**D**) *COX-2*, (**E**) *TNF-α*, (**F**) *IFN-γ*, and (**G**) *TLR-4* expression. There was significant difference between the CY group (*, *p* < 0.05) and ginseng group (#, *p* < 0.05).

CY treatment significantly suppressed the expression of immune-associated genes including *IL-1β*, *IL-6*, *TNF-α*, *IFN-γ*, *COX-2*, *iNOS*, and *TLR-4* genes in peritoneal macrophages (Fig. 3). Compared to the CY-treated group, the mixture of CFAM and red ginseng extract highly increased the expression of these immune-associated genes. In addition, peritoneal macrophages treated with 25 and 50 mg/kg BW of CFAM plus 100 mg/kg BW of red ginseng extract showed higher expression of these immune-associated genes than the red ginseng alone-treated group. Especially, the mixture of CFAM and red ginseng extract showed even higher response of immune-associated genes in peritoneal macro-phages than in normal mice.

## Discussion

Macrophages are immune cells that play critical roles in resisting infection and eliminating tumor cells in the host [37]. They secrete NO that contributes to the immune response [38] and whose main function is to mediate the ability of the activated macrophage to kill bacteria and tumor cells [39] i.e., macrophages protect the host cells from infection by increasing the production of NO [40]. Phagocytosis is one of the first defense mechanisms against infection by pathogens [12, 41] and is also crucial for both adaptive and innate immunity [42]. Macrophages are one of the most important types of cells among the phagocytes in our immune system [24]. Fig. 1 shows that CFAM and red ginseng extract enhanced the viability and NO production of peritoneal macrophages, whereas CY suppressed peritoneal macrophage viability and NO production. In addition, CFAM and red ginseng extract increased the phagocytosis activity of peritoneal macrophages, suggesting that CFAM and red ginseng extract enhanced non-specific immune functions in the immune-suppressed mice.

Macrophages play critical roles in innate and adaptive immune responses to pathogens through phagocytosis, antigen presentation, and cytokines secretion [43]. In these cells, the iNOS gene regulates the production of NO which is a well-known pro-inflammatory mediator [44] and secrete pro-inflammatory cytokines such as TNF-α and IL-1β [45]. The secreted cytokines are important for antigen defense as they regulate the immune system for anti-bacterial [46], anti-viral [47] and anti-tumor functions [48]. As shown in Fig. 3, CY suppressed the cytokine expression levels of peritoneal macrophages compared to the normal mice. However, the treatment of CFAM and red ginseng extract enhanced peritoneal macrophage cytokine expression responses to B cell mitogen (LPS). This indicates that CFAM and red ginseng extract could promote the secretion of peritoneal macrophage cytokines in order to restore immunosuppression.

The present study demonstrated that CFAM mixed with red ginseng extract enhanced the immune responses in CY-induced immune-suppressed mice through peritoneal macrophage viability, NO production, phagocytosis of peritoneal macrophage, and immune-associated gene expre-ssion in peritoneal macrophage. These immune response activities of CFAM mixed with red ginseng extract showed higher activities than those observed in the red ginseng only treated-group. CFAM enhanced the immune activity of red ginseng extract in the peritoneal macrophage cells of immune-suppressed mice. Furthermore, CFAM might be used as a co-stimulant of red ginseng extract through the regulation of macrophage cells for the enhancement of human health and immunity.

## Figures and Tables

**Table 1:** Oligonucleotide primers used for evaluating immune gene.

Gene	Accession No.	Sequences
*IL-1β*	NM_008361.4	Forward: GGGCCTCAAAGGAAAGAATC
		Reverse: TACCAGTTGGGGAACTCTGC
*IL-6*	NM_031168.2	Forward: AGTTGCCTTCTTGGGACTGA
		Reverse: CAGAATTGCCATTGCACAAC
*IFN-γ*	NM_008337.3	Forward: CTCAAGTGGCATAGATGT
		Reverse: GAGATAATCTGGCTCTGCAGGATT
*TNF-α*	D84199.2	Forward: ATGAGCACAGAAAGCATGATC
		Reverse: TACAGGCTTGTCACTCGAATT
*TLR-4*	NM_021297.3	Forward: CGCTCTGGCATCATCTTCAT
		Reverse: GTTGCCGTTTCTTGTTCTTCC
*COX-2*	NM_011198.4	Forward: AGAAGGAAATGGCTGCAGAA
		Reverse: GCTCGGCTTCCAGTATTGAG
*iNOS*	BC062378.1	Forward: TTCCAGAATCCCTGGACAAG
		Reverse: TGGTCAAACTCTTGGGGTTC
*β-actin*	NM_007393.5	Forward: CCACAGCTGAGAGGGAAATC
		Reverse: AAGGAAGGCTGGAAAAGAGC
